# Supplementary material for: Review: Influence of 25(OH)D Blood Concentration and Supplementation during Pregnancy on Preeclampsia Development and Neonatal Outcomes
Source: Int J Mol Sci. 2022 Oct 26;23(21):12935. doi: 10.3390/ijms232112935 (PMC9653937; doi:10.3390/ijms232112935)
Supplement: Supplementary file 1 [file ijms-23-12935-s001.zip › ijms-1979356-supplementary.pdf]

Supplementary Materials

In the mentioned studies for table 1 and 2 the 25(OH)D concentration measurements were performed with various methods like ELISA, ECLIA, Chemiluminescent Assay, Liquid Chromatography coupled with Mass Spectrometry. Some of these methods like ELISA could give cross-reaction between 25(OH)D2 and 25(OH)D3 and could not distinguish these two forms In initial HPLC validation, they observed that <10% samples had any measurable 25(OH)D2, and within these samples, 25(OH)D2 accounted for only 10% of the total measurable 25(OH)D [11]. The difference between the kits and analysis equipment for chemiluminescent assays could also influence the precision of the obtained results. In the supplemental material section the equivalent tables are presented with information about the laboratory measurement of 25(OH)D and DEQUAS International Quality Control.

Table S1. Laboratory Method of Measurement of 25(OH)D concentrations during normal and complicated by preeclampsia pregnancy.

Laboratory Method of Measurement	DEQUAS	References
First trimester of gestation (< 14)		
Chemiluminescent Assay Diasorin	-	[44]
Chemiluminescent Assay Diasorin	+	[48]
First–second trimester of gestation		
Chemiluminescent Assay Diasorin	+	[47]
Liquid Chromatography Mass Spectrometry	-	[52]
Second trimester of gestation (15-26)		
Liquid Chromatography Mass Spectrometry	-	[31]
Liquid Chromatography Mass Spectrometry	-	[54]
ELISA	-	[11]
Chemiluminescent Assay Diasorin	+	[49]
Second–third trimester of gestation		

Liquid Chromatography Mass Spectrometry	-	[57]
Chemiluminescent Assay Diasorin	-	[27]
Chemiluminescent Assay Diasorin	-	[43]
Liquid Chromatography Mass Spectrometry	-	[53]
Third trimester of gestation (> 26)		
Chemiluminescent Assay Diasorin	-	[30]
Chemiluminescent Assay Diasorin	-	[20]
Chemiluminescent Assay Diasorin	-	[15]
Chemiluminescent Assay Diasorin	-	[14]
ELISA	-	[22]
Radioimmunoassay	-	[28]
Liquid Chromatography Mass Spectrometry	-	[52]
Chemiluminescent Assay Diasorin	+	[47]
ECLIA	-	[35]
ELISA	-	[19]

Table S2. Laboratory Method of Measurement of 25(OH)D concentrations in newborn with negative neonatal and postnatal outcomes born to mothers with preeclampsia

Laboratory Method of Measurement	DEQUAS	Reference
Neonatal Outcomes		
Liquid Chromatography Mass Spectrometry	+	[80]
Liquid Chromatography Mass Spectrometry	+	[50]
Chemiluminescent Assay Diasorin	-	[97]
Liquid Chromatography Mass Spectrometry	-	[99]
-	-	[100]
Postnatal Outcomes		
Chemiluminescent AssayModular E170 analyzer	-	[98]
